# PearMODB: a multiomics database for pear (*Pyrus*) genomics, genetics and breeding study

**DOI:** 10.1093/database/baad050

**Published:** 2023-07-06

**Authors:** Jian Hu, Baisha Huang, Hao Yin, Kaijie Qi, Yuanyuan Jia, Zhihua Xie, Yuan Gao, Hongxiang Li, Qionghou Li, Zewen Wang, Ying Zou, Shaoling Zhang, Xin Qiao

**Affiliations:** Sanya Institute of Nanjing Agricultural University, State Key Laboratory of Crop Genetics & Germplasm Enhancement and Utilization, Nanjing Agricultural University, No.1 Weigang, Nanjing 210095, China; Jiangsu Engineering Research Center for Pear, Nanjing Agricultural University, Nanjing 210095, China; College of Horticulture, Nanjing Agricultural University, Nanjing 210095, China; Sanya Institute of Nanjing Agricultural University, State Key Laboratory of Crop Genetics & Germplasm Enhancement and Utilization, Nanjing Agricultural University, No.1 Weigang, Nanjing 210095, China; Jiangsu Engineering Research Center for Pear, Nanjing Agricultural University, Nanjing 210095, China; College of Horticulture, Nanjing Agricultural University, Nanjing 210095, China; Sanya Institute of Nanjing Agricultural University, State Key Laboratory of Crop Genetics & Germplasm Enhancement and Utilization, Nanjing Agricultural University, No.1 Weigang, Nanjing 210095, China; Jiangsu Engineering Research Center for Pear, Nanjing Agricultural University, Nanjing 210095, China; College of Horticulture, Nanjing Agricultural University, Nanjing 210095, China; Sanya Institute of Nanjing Agricultural University, State Key Laboratory of Crop Genetics & Germplasm Enhancement and Utilization, Nanjing Agricultural University, No.1 Weigang, Nanjing 210095, China; Jiangsu Engineering Research Center for Pear, Nanjing Agricultural University, Nanjing 210095, China; College of Horticulture, Nanjing Agricultural University, Nanjing 210095, China; Sanya Institute of Nanjing Agricultural University, State Key Laboratory of Crop Genetics & Germplasm Enhancement and Utilization, Nanjing Agricultural University, No.1 Weigang, Nanjing 210095, China; Sanya Institute of Nanjing Agricultural University, State Key Laboratory of Crop Genetics & Germplasm Enhancement and Utilization, Nanjing Agricultural University, No.1 Weigang, Nanjing 210095, China; Jiangsu Engineering Research Center for Pear, Nanjing Agricultural University, Nanjing 210095, China; College of Horticulture, Nanjing Agricultural University, Nanjing 210095, China; College of Horticulture, Nanjing Agricultural University, Nanjing 210095, China; Sanya Institute of Nanjing Agricultural University, State Key Laboratory of Crop Genetics & Germplasm Enhancement and Utilization, Nanjing Agricultural University, No.1 Weigang, Nanjing 210095, China; Jiangsu Engineering Research Center for Pear, Nanjing Agricultural University, Nanjing 210095, China; College of Horticulture, Nanjing Agricultural University, Nanjing 210095, China; Sanya Institute of Nanjing Agricultural University, State Key Laboratory of Crop Genetics & Germplasm Enhancement and Utilization, Nanjing Agricultural University, No.1 Weigang, Nanjing 210095, China; Jiangsu Engineering Research Center for Pear, Nanjing Agricultural University, Nanjing 210095, China; College of Horticulture, Nanjing Agricultural University, Nanjing 210095, China; Sanya Institute of Nanjing Agricultural University, State Key Laboratory of Crop Genetics & Germplasm Enhancement and Utilization, Nanjing Agricultural University, No.1 Weigang, Nanjing 210095, China; Jiangsu Engineering Research Center for Pear, Nanjing Agricultural University, Nanjing 210095, China; College of Horticulture, Nanjing Agricultural University, Nanjing 210095, China; Sanya Institute of Nanjing Agricultural University, State Key Laboratory of Crop Genetics & Germplasm Enhancement and Utilization, Nanjing Agricultural University, No.1 Weigang, Nanjing 210095, China; Jiangsu Engineering Research Center for Pear, Nanjing Agricultural University, Nanjing 210095, China; College of Horticulture, Nanjing Agricultural University, Nanjing 210095, China; Sanya Institute of Nanjing Agricultural University, State Key Laboratory of Crop Genetics & Germplasm Enhancement and Utilization, Nanjing Agricultural University, No.1 Weigang, Nanjing 210095, China; Jiangsu Engineering Research Center for Pear, Nanjing Agricultural University, Nanjing 210095, China; College of Horticulture, Nanjing Agricultural University, Nanjing 210095, China; Sanya Institute of Nanjing Agricultural University, State Key Laboratory of Crop Genetics & Germplasm Enhancement and Utilization, Nanjing Agricultural University, No.1 Weigang, Nanjing 210095, China; Jiangsu Engineering Research Center for Pear, Nanjing Agricultural University, Nanjing 210095, China; College of Horticulture, Nanjing Agricultural University, Nanjing 210095, China

## Abstract

Pear (*Pyrus* ssp.) belongs to Rosaceae and is an important fruit tree widely cultivated around the world. Currently, challenges to cope with the burgeoning sets of multiomics data are rapidly increasing. Here, we constructed the Pear Multiomics Database (PearMODB) by integrating genome, transcriptome, epigenome and population variation data, and aimed to provide a portal for accessing and analyzing pear multiomics data. A variety of online tools were built including gene search, BLAST, JBrowse, expression heatmap, synteny analysis and primer design. The information of DNA methylation sites and single-nucleotide polymorphisms can be retrieved through the custom JBrowse, providing an opportunity to explore the genetic polymorphisms linked to phenotype variation. Moreover, different gene families involving transcription factors, transcription regulators and disease resistance (nucleotide-binding site leucine-rich repeat) were identified and compiled for quick search. In particular, biosynthetic gene clusters (BGCs) were identified in pear genomes, and specialized webpages were set up to show detailed information of BGCs, laying a foundation for studying metabolic diversity among different pear varieties. Overall, PearMODB provides an important platform for pear genomics, genetics and breeding studies.

**Database URL** http://pearomics.njau.edu.cn

## Introduction

Pear (*Pyrus*, 2*n* = 2*x* = 34), belonging to the Rosaceae family and the Maloideae subfamily (1), is an important fruit crop and has been widely cultivated in China. There are >22 described species in the genus *Pyrus*, among which five major species (*P. pyrifolia, P. bretschneideri, P. ussuriensis, P. × sinkiangensis* and *P. communis*) have been domesticated to comprise the majority of modern cultivated varieties. As of 2021, the pear-cultivated area around the world has reached 1 399 484 ha, and the yield is ∼2 568 713.07 tons (http://www.fao.org/faostat/en/#home).

In the year 2013, the first pear genome (*P. bretschneideri* Rehd. cv. ‘Dangshansuli’, Asian pear) was published with 512 Mb assembly size and 42 812 annotated genes, and genome evolution and important genes related to pear fruit quality (e.g. sugar, aroma and stone cell) were resolved (2). Meanwhile, a data portal for accessing the pear genome dataset was built (http://peargenome.njau.edu.cn), but limited data and web services were provided. In the year 2014, the first genome representing European pear (*P. communis* cv. ‘Bartlett’) was published (3) and has been updated to a higher-contiguity chromosome-level assembly in 2019 (‘Bartlett DH v2.0’) with assembly size 445 Mb and predicted genes 37 445 (4). In recent years, high-quality genomes of several other pear varieties have been reported, including wild pear ‘Shanxi Duli’ published in 2020 (5), dwarf pear ‘Zhongai 1’ in 2019 (6), Japanese pear ‘Nijisseiki’ (7), Chinese sand pear ‘Cuiguan’ in 2021 (8) and European pear ‘d’Anjou’ in 2022 (9). In addition, haplotype assembly for diploid ‘Dangshansuli’ has been achieved based on single-pollen-cell sequencing (10). However, current efforts to pear genome assembly remain limited given to >5000 described pear accessions around the world (11). The considerable challenges are rising for storing, accessing and analyzing huge volumes of 5000 pear genomes.

The reference genome assembly of pear has promoted population genomics studies. Genome resequencing of 113 worldwide pear accessions revealed abundant genetic variation among wild and cultivated pear species, with a total of 18 302 883 single-nucleotide polymorphisms (SNPs) identified, and 9.29 and 5.35Mb genome regions for Asian pears and European pears were found to be influenced by domestication, respectively (11). Moreover, 3.4 million SNPs were discovered based on genome resequencing of 312 sand pear varieties, and 11.1 Mb genome region showed a signature of selective sweeps harboring 1417 genes (12). Analysis of ribonucleic acid (RNA)-seq data from fruit flesh samples of 206 pear cultivars collected at 49 days after full bloom revealed 974 404 SNPs and 139 515 expression quantitative trait loci (13). In addition, 15 000 SNPs were identified from 214 pear accessions based on the genotyping-by-sequencing method (14). The large-scale population variation data are highly desired to be integrated to accelerate trait-related gene loci identification, bridging gaps between genetic polymorphism and phenotype diversity.

Transcriptome sequencing involving different pear tissues, different development stages and under different conditions has been extensively conducted over past decades. The available transcriptome data for pear provide a valuable resource for refining gene annotation, studying gene expression patterns, inferring gene regulatory networks and resolving gene function. By the end of the year 2022, 1422 transcriptomes involved in pear have been deposited in the Sequence Read Archive (SRA) of National Center for Biotechnology Information (NCBI) database (https://www.ncbi.nlm.nih.gov/sra), including those from different pear tissues (15), different development stages and different varieties (16), various treatments (17) and different conditions (18). Currently, the wave of 3D-genome, epigenome, proteome, spatiotemporal single-cell RNA and metabolome sequencing will generate large-scale omic datasets for pear. Therefore, a comprehensive database to integrate different types of omics data and include online tools to meet diverse demands for data analysis is highly desired in the multiomics era.

Here, we constructed the Pear Multiomics Database (PearMODB) (http://pearomics.njau.edu.cn) based on the integration of different types of omics data. A variety of online tools were developed for quickly accessing and analyzing pear multiomics data, including tools for gene search, sequence alignment, multifaceted genomic information browser, gene family search, transcriptional expression heatmap and synteny analysis. Particularly, biosynthetic gene clusters (BGCs) were identified at a genome-wide scale for seven pear varieties. The PearMODB will accelerate the integration of available pear multiomics data and facilitate future studies in pear genomics, genetics and breeding.

## Materials and methods

### Data source

PearMODB hosted genome assembly and gene annotation data for seven pear varieties. Two haplotype assemblies and gene annotations of diploid ‘Dangshansuli’ are also available (10). The diploid mosaic assembly and gene annotation of ‘Dangshansuli’ were previously published by our research group (http://peargenome.njau.edu.cn) (2, 10). Genome data of ‘Bartlett’ were downloaded from GigaDB (http://www.gigadb.org/) (4). ‘Nijisseiki’ and ‘D’Anjou’ genome data were obtained from the Genome Database for Rosaceae (GDR) (https://www.rosaceae.org) (7, 9). ‘Cuiguan’ and ‘Duli’ genome data were downloaded from Genome Warehouse (https://ngdc.cncb.ac.cn/gwh/) (5, 8). ‘Zhongai 1’ genome data were downloaded from Figshare (https://figshare.com/) (6). RNA-seq data of 269 pear transcriptomes from different varieties, different tissues, different development stages and conditions were downloaded from NCBI SRA (https://www.ncbi.nlm.nih.gov/sra). Population variation data (SNPs) were obtained from a previous study (https://www.ncbi.nlm.nih.gov/bioproject/?term=PRJNA381668) (11). DNA methylation data were downloaded from NCBI (https://www.ncbi.nlm.nih.gov/bioproject/?term=PRJNA503323) (15). The detailed information about the source of data can be retrieved from Supplementary Table S1.

### Quantification of gene expression abundance

Trimmomatic (19) was used to remove adapter sequences and poly(A/T) tails and filter low-quality reads (quality score <15) from raw RNA-seq reads. Kallisto (20) was used to estimate the abundance of transcripts per million (TPM) with default parameters.

### Gene function annotation and collinearity analysis

eggNOG (21) was used to predict gene function, specifying the diamond method using the -m parameter and setting the seed_ortholog_evalue to 1e^−5^. For collinearity analysis, first pairwise BLAST of whole-genome protein sequences between any two pear varieties and within each variety was performed using diamond software with an *e*-value cutoff of 1e^–10^ (22). Then, the BLAST results and chromosomal location information of genes were used as input files for MCScanX software to generate a collinearity file (23). The DupGen_Finder pipeline was utilized to identify gene pairs derived from different modes of gene duplication events using sacred lotus (*Nelumbo nucifera*) as an outgroup (24).

### Identification and classification of gene families

We used iTAK software (25) to identify and classify transcription factors (TFs), transcriptional regulators (TRs) and protein kinases (PKs) families from seven pear genomes. Identification and classification of TFs and TRs were based on the rules that have been widely adopted in PlnTFDB (26), PlantTFDB (27), PlantTFcat (28) and AtTFDB (29). PKs were identified based on specific domains corresponding to 16 PKinase clans (30).

We downloaded the NB-ARC (nucleotide-binding adaptor shared by APAF-1, R proteins, and CED-4) domain (PF00931) sequence from the Pfam database. HMMER (31) software was used to search candidate nucleotide-binding site (NBS)-encoding genes with a threshold expectation value of 1, and BLASTP search was also performed to identify the candidate NBS-encoding genes (*e*-value: <1e^−20^) in seven pear genomes using previously reported *Arabidopsis* NBS-encoding genes as query sequence (32). Then, NCBI–Conserved Domain Database (CDD) (https://www.ncbi.nlm.nih.gov/cdd/) was used to identify the NBS structure domain. Finally, amino acid sequences of candidate NBS-encoding genes were submitted to NCBI-CDD to identify toll interleukin 1 receptor and leucine-rich repeat (LRR) domains. The COILS program was used to specifically detect the coiled-coil domains in candidate NBS-encoding genes at a threshold of 0.9 (33).

## Results

### Database construction

**Figure 1.**
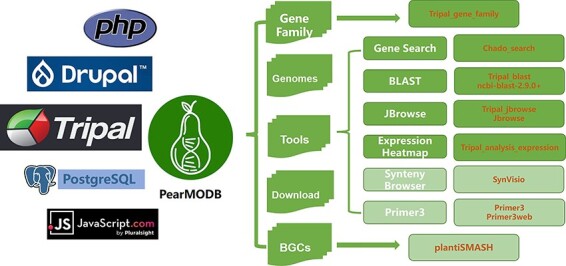
The framework and available function modules of PearMODB. Core system and programming language, logo, main functional modules, corresponding core programs of PearMODB are shown from left to right.

PearMODB is developed based on Tripal (http://tripal.info/), which is a toolkit for constructing online genomic and genetic databases. The web pages of PearMODB are designed and established based on HTML+CSS+JavaScript and managed by Drupal (a website management system based on the PHP programming language). The data are loaded using a GMOD/Chado database schema (34–36). A variety of online tools are built by embedding various extension modules developed by the Tripal community and external tools (Figure 1).

### Database introduction

#### The home page of PearMODB

**Figure 2.**
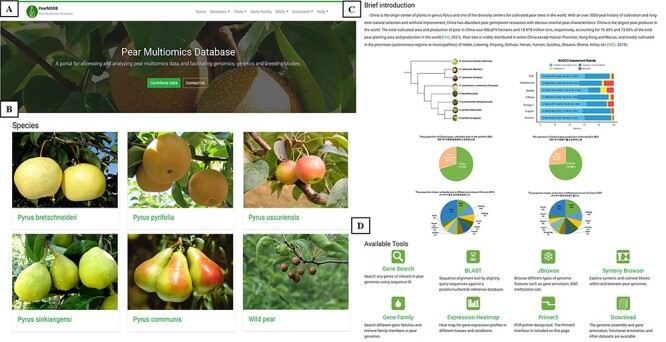
The home page of PearMODB. (**A**) Drop-down menus to explore different function modules and brief introduction. (**B**) Representative cultivars of five major cultivated species and wild pear species. (**C**) Brief introduction about pear cultivation and yield. (**D**) Available tools on PearMODB.

The home page of PearMODB contains a horizontal menu bar at the top right of the page, a brief introduction of database, a presentation of different pear varieties and available tools (Figure 2). The menu bar contains seven drop-down menus: Home, Genomes, Tools, Gene Family, BGCs, Download and Help (Figure 2A). The menu bar allows the user to find and interact with various function modules implemented in PearMODB, including searching gene sequences, browsing genomic features, visualizing gene expression profiles, downloading genome data and other essential functions. The images correspond to five major cultivated pear species and wild pear species, and the call-to-action button allows users to quickly jump to a new page to explore detailed information of representative varieties including main characteristics and fruit pictures of each variety (Figure 2B). In addition, the phylogenetic tree of seven sequenced pear varieties, quality of genome annotation and information about pear cultivation area and yield in China and around the world are presented (Figure 2C). Users could also quickly jump to the main page of all available tools by clicking on the tool icon of interest (Figure 2D).

#### Species page

At present, genomic data for seven pear varieties have been integrated into PearMODB (Table 1; Supplementary Table S1). The sequencing platform, genome size, gene number, assembly quality and data source for published pear genomes were presented. A vertical menu bar located at the left of the page contains five menus: Overview, Publication, Download, JBrowse and BLAST. The ‘Overview’ page provides detailed information for each pear accession, including genome sequencing, assembly and annotation information. The ‘Publication’ page showed publication information for the genome report. The ‘Download’, ‘JBrowse’ and ‘BLAST’ functions allow users to quickly jump to the corresponding pages to download genomic data, browse genomic features and search for homologous sequences.

**Table 1.** Genome assembly and gene annotation information for seven pear varieties

Varieties	Estimated size (Mb)	Assembly size (Mb)	Sequencing platforms	Gene number	Contig N50 (Mb)	Scaffold N50 (Mb)	Data source
Dangshansuli	527	512	Illumina	42 812	0.04	0.5	Pear Genome Project
Bartlett	528	445	PacBio+Bionano+Hi-C	37 445	5.3	6.5	GigaDB
Nijisseiki	537	504	PacBio	44 876	7.6	31.6	GDR
Cuiguan	501	541	PacBio+Illumina+Hi-C	42 257	1.3	28.0	Genome Warehouse
Duli	511	533	PacBio+Bionano+Hi-C	59 552	1.6	28.1	Genome Warehouse
Zhongai 1	511	511	PacBio+Illumina+Hi-C	43 120	1.3	23.5	Figshare
D’Anjou	NA	600	PacBio+Illumina	45 981	NA	0.4	GDR

#### Available tools

##### Gene search

**Figure 3.**
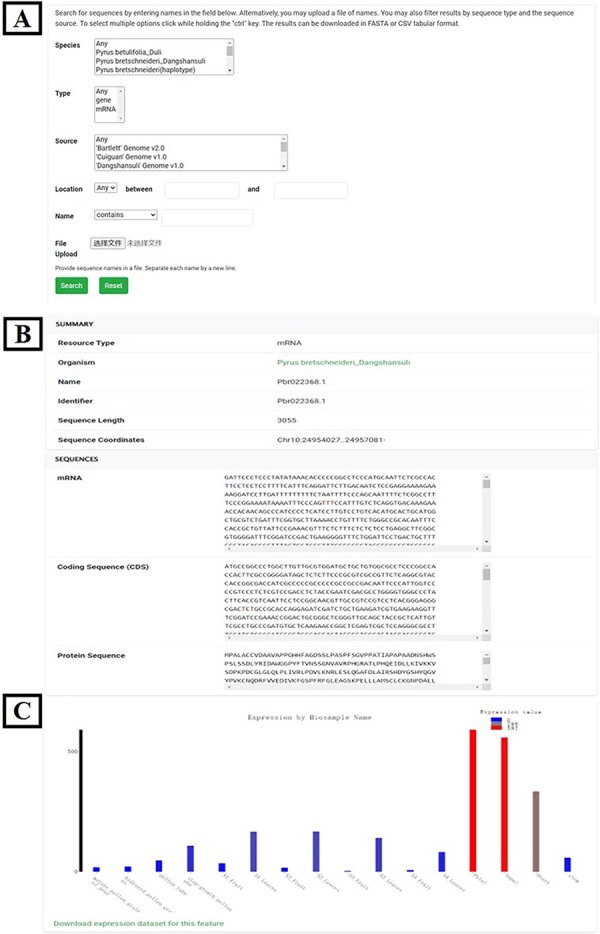
Gene search tool. (**A**) Gene search page. (**B**) Chromosomal location and sequences of the target gene. (**C**) Spatiotemporal expression profile of target gene.

A total of 382 662 annotated genes from seven pear varieties, including their nucleotide and protein sequences, were loaded into the local Chado database using the ‘chado_search’ module (https://gitlab.com/mainlabwsu/chado_search). Flexible options were provided on this page to enable specific gene search for users (Figure 3A). The chromosomal location, sequence length, CDS, protein sequence (Figure 3B) and spatiotemporal expression profile (Figure 3C) will be returned for target sequence IDs inputted or uploaded by users.

##### BLAST

**Figure 4.**
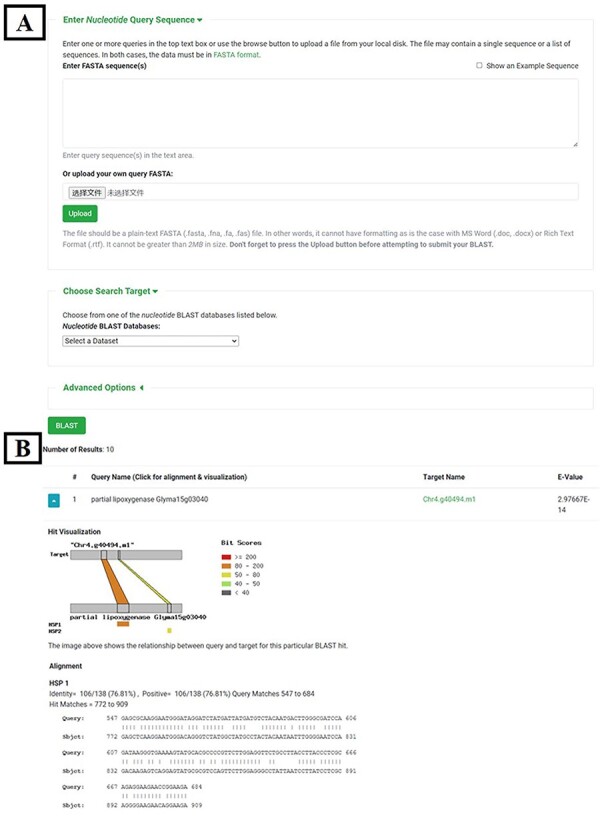
BLAST tool. (**A**) BLAST search page. (**B**) BLAST result page.

Ncbi-blast-2.9.0+ (32) was used to create local BLAST database files from genome sequences, CDS and protein sequences, respectively, of seven sequenced pear varieties. The ‘tripal_blast’ (https://github.com/tripal/tripal_blast) module was used to build an interface for the BLAST search (Figure 4A). The result page of the BLAST search is an expandable summary table with each hit being listed as a row in the table, containing query sequence ID, subject sequence ID and *e*-value (Figure 4B). The row for each hit pair can be unfolded to show detailed alignment information including hit visualization and high-scoring pair between a query and subject sequence. Different formats of BLAST search results, including BLAST pairwise format, BLAST tabular format, GFF3 and BLAST XML format, are available to download.

##### Genome browser

**Figure 5.**
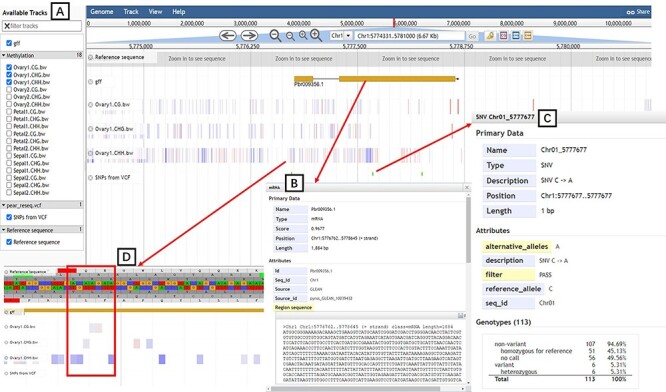
JBrowse tool. (**A**) Available tracks for different types of genomic features. (**B**) A window showing detailed information of the target gene model. (**C**) SNPs information for 113 pear accessions. (**D**) Tracks of three types of DNA methylation.

JBrowes is a genome browser that supports interactive access and view for different types of genomic features (37). The ‘tripal_jbrowse’ (https://github.com/tripal/tripal_jbrowse) module was utilized to build a custom JBrowse for each of the seven pear varieties (Figure 5). The tracks of reference genome sequences and gene models are shown on the JBrowse page to provide a graphical and informative view of chromosomal location, gene structure and sequences (Figure 5B). In particular, SNPs identified from genome resequencing data of 113 pear accessions and DNA methylation data have been added to the JBrowse page of ‘Dangshansuli’ genome (Figure 5D; Supplementary Table S1).

The SNP and different types of DNA methylation sites for any locus of interest can be browsed through the custom JBrowse for cv. ‘Dangshansuli’. For example, we explored the SNPs and DNA methylation sites located in gene Pbr009356.1. We can view the gene position, gene length and sequence by clicking the gene model (Figure 5B). By clicking one SNP site (Figure 5C), we can view the chromosome location, variation type and frequency of the site among 113 pear accessions (Figure 5C). By enlarging the window size of JBrowse, we can view three types of DNA methylation and level of methylation at each site (Figure 5D).

##### Expression Heatmap

**Figure 6.**
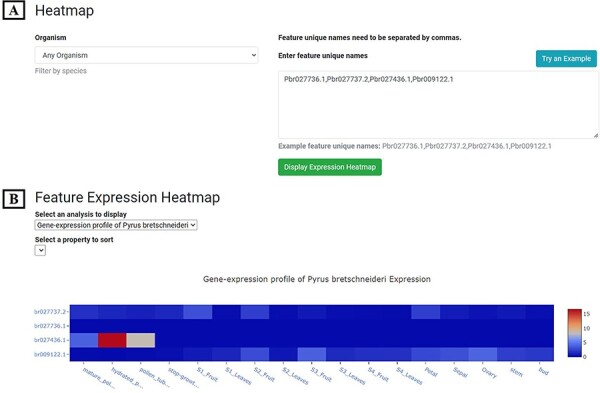
Expression Heatmap page. (**A**) The block for organism selection and gene id input. (**B**) Gene expression profile of example genes in 17 tissues or development stages of *P. bretschneideri* cv. ‘Dangshansuli’.

A total of 269 transcriptomes from different pear tissues, different development stages and different conditions were collected and integrated into PearMODB (Supplementary Table S1). ‘Expression Heatmap’ was built using the extension module ‘tripal_expression_analysis’ (https://github.com/tripal/tripal_analysis_expression). ‘Expression Heatmap’ allows users to retrieve spatiotemporal expression profiles of any genes of interest (Figure 6).

##### Synteny Browser

**Figure 7.**
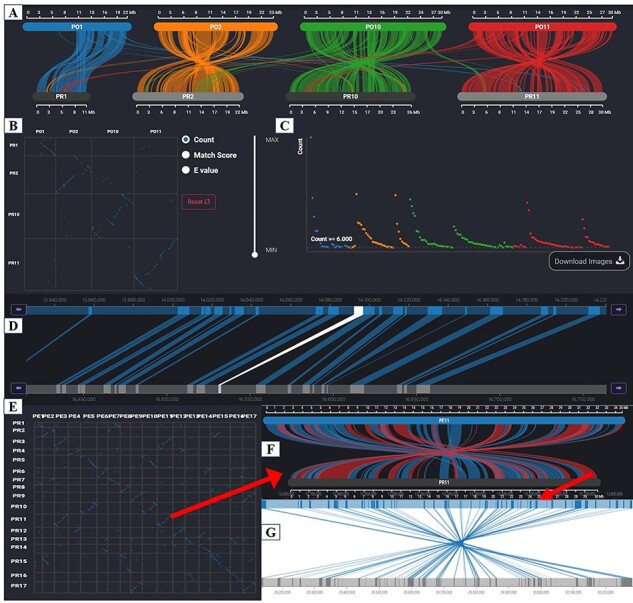
Synteny Browser page. (**A**) Macrosynteny visualization between the two pear variety genomes. (**B**) Dot plot corresponding to the macrosynteny visualization. (**C**) Distribution of the number of syntenic blocks with different lengths. (**D**) Microsynteny visualization of the target gene. (**E**) Dot plot of collinearity relationships of 17 chromosomes between ‘Dangshansuli’ and ‘Duli’. (**F**) Macrosynteny visualization of Chr11 between ‘Dangshansuli’ and ‘Duli’. (**G**) Microsynteny visualization of the selected syntenic block.

‘Synteny Browser’ was built by embedding the SynVisio interface (https://github.com/kiranbandi/synvisio) and allowed users to explore syntenic blocks within and between genomes of different pear varieties. The multiscale macro- and microsynteny visualization tools were available. Users could retrieve syntenic blocks for any gene ID of interest and browse gene pairs contained in the target syntenic block (Figure 7).

‘Synteny Browser’ can be used to detect chromosome structural variations such as insertions, deletions, inversions and translocations and identify conserved chromosomal regions between species. Figure 7E shows the genome-wide collinear relationships between ‘Dangshansuli’ and ‘Duli’, and many chromosomal inversions between these two varieties were observed. Chromosome 11 between two varieties was zoomed in to view inverted segments and paralleled segments (Figure 7F). We can view genes located in any syntenic blocks by clicking on red or blue bands (Figure 7G).

##### Primer3

To provide web services for polymerase chain reaction (PCR) primer design, the primer3web interface (https://github.com/primer3-org/primer3web) was embedded into PearMODB (38). The Primer3 page allows users to enter any gene sequence of interest and obtain PCR primers for gene cloning. Moreover, users can perform custom primers design by setting different parameters on this page, such as the size of the primer and the guanine-cytosine content of the primer.

#### Gene Family

TFs are proteins that bind to motifs contained in the promoters of the target gene to control its transcription. TRs indirectly regulate the expression of target genes by interacting with TFs (28). TFs and TRs play important roles in plant development and stress response. We identified TF, TR and PK family genes in the genomes of seven pear varieties (Table 1) and classified them into different subfamilies using iTAK software (25). On this page, users can browse all gene members in any gene family of interest.

**Figure 8.**
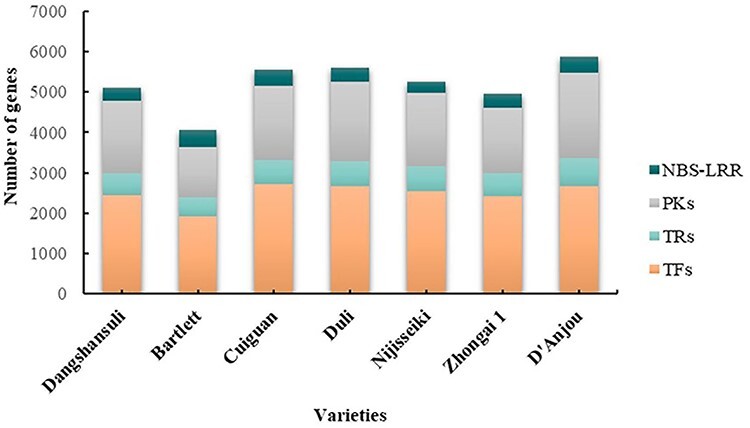
A comparison of gene number of different families in seven pear genomes.

NBS-LRR family genes are involved in the response of the plant’s innate immune system to pathogen infection and play an important role in defense against pathogens (39). The NBS-LRR family in seven pear varieties’ genomes was identified and classified into different subfamilies and integrated into PearMODB (Figure 8).

#### BGCs

**Figure 9.**
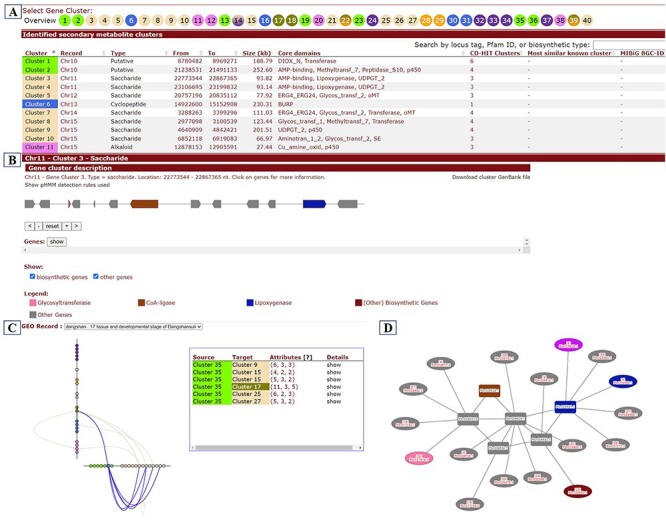
BGCs page. (**A**) Whole set of BGCs in a pear genome. (**B**) Gene members and functional characteristics of BGCs. (**C**) Co-expression relationships between BGCs. (**D**) Co-expression network of genes within a selected cluster (rectangle) and their co-expression relationships with genes from other clusters (ellipse).

BGCs participate in the biosynthesis of specialized metabolites and play an important role in plant growth and development and stress response. BGCs were identified from seven pear genomes (Table 1) using plantiSMASH software and classified into seven types: saccharide, polyketide, cyclopeptide, terpene, alkaloid, lignan and putative (40). The custom webpages have been built to show detailed information of BGCs, including chromosomal location, gene members for each BGC, expression patterns of BGCs and co-expression profiles. (Figure 9). The whole set of BGCs in each of the pear genomes was presented as that in Figure 9A. Gene members and their functional types for each BGC were shown as that in Figure 9B. Based on transcriptome data of 17 different tissues and developmental stages of ‘Dangshansuli’ (Supplementary Table S1), we constructed the co-expression network and determined co-expression relationships within and between BGCs, which can be viewed on this page (Figure 9C and D).

#### Download

‘Download’ contains four drop-down menus, including Genome Data, Family Genes Sequences, Duplicated Gene Pairs and Functional Annotation. The genome assembly and gene annotation files (gff, CDS and protein) are available on the ‘Genome Data’ page. The protein sequences of TF, TR, PK and NBS-LRR family genes are available on ‘Family Genes Sequences’. We have also identified different modes of duplicated genes in genomes of seven pear varieties using DupGen_finder software (24), and the results can be accessed on the ‘Duplicated Gene Pairs’ page. In addition, we have performed functional annotation for whole-genome genes in each of the seven pear varieties using eggNOG software (21), and functional annotation files can be downloaded from the ‘Functional Annotation’ page.

## Discussion

PearMODB was developed to provide a comprehensive platform to access and analyze pear (*Pyrus*) multiomics data. PearMODB integrated different types of pear omics data and developed various online tools to cope with increasing requirements for diversified data analysis. Currently, PearMODB includes genome assembly, gene annotation, functional annotation, transcriptome, DNA methylation and population variation data. Available tools include gene search, BLAST, genome browser, expression heatmap, synteny browser and primer design. TF, TR, PK and NBS-LRR families are available for gene members search, providing a reference for gene function study and comparative genomics analysis. Different modes of duplicated gene pairs identified in seven pear genomes are also available on PearMODB. Specially, PearMODB provides an opportunity for investigating BGCs.

Three genome databases involving pear have been built, including GDR (https://www.rosaceae.org) (41), Pear Genome Project (http://peargenome.njau.edu.cn) (2) and PearEXP (http://www.peardb.org.cn/) (42). The GDR hosts genome assembly and gene annotation data of seven pear varieties but does not have the haplotype assembly and annotation data of cv. ‘Dangshansuli’ that are available on PearMODB. Compared with GDR, PearMODB possesses several special features. First, PearMODB provides the ‘Expression Heatmap’ tool to explore the spatiotemporal expression profile of genes of interest based on the integration of 269 transcriptomes from different pear varieties and conditions. Second, PearMODB integrated population variation data from genome resequencing of 113 pear germplasms (11) and DNA methylation data from different tissues (15). Third, PearMODB provides the ‘Gene Family’ module to search gene members of different gene families. Particularly, the information of BGCs, such as chromosomal location, gene members and expression patterns, is available on PearMODB for studying metabolic differences among different pear varieties. Pear Genome Project was built previously by our research team, which provided only web service for downloading genome data of cv. ‘Dangshansuli’ (2). PearEXP database was constructed recently by our research team (42), which is mainly used to present pear proteome data, and also includes function modules to survey gene expression profiles. However, less transcriptome data were incorporated in PearEXP in contrast with PearMODB. The transcriptome data in PearEXP were from only one variety cv. ‘Dangshansuli’. Nonetheless, visualization of transcriptome or proteome in PearEXP is superior to that in PearMODB. In future versions of PearMODB, we will make efforts to upgrade the visualization tools involved in gene expression profiles and integrate proteome data into PearMODB to provide a multiomics platform from a single resource for user convenience.

The future efforts would be made to exploit function modules involved in genome-wide association analysis between genetic polymorphisms and phenotypes, bridging gaps between genotype and phenotype. Large-scale population variation data such as SNP, copy-number variation and structural variation will be integrated to accelerate trait-related gene loci identification. Moreover, the construction of a gene co-expression network and a gene regulation network based on the integration of multiomics data will provide an important basis for the accurate prediction of gene function and the identification of molecular regulation pathways of important traits in pear.

Driving by the rapid development of sequencing technology, large-scale omics data involved in pear pangenome, 3D-genome, proteome, metabolome, phenome and single-cell sequencing data will be available in the foreseeable future. PearMODB will be updated as more omics data become available and new tools for data analysis and visualization will be exploited. Collectively, PearMODB will be an important data hub for pear genomics, gene function and breeding studies.

## Supplementary Material

baad050_Supp

## Data Availability

All data described in this manuscript is available at http://pearomics.njau.edu.cn, please refer to Supplementary material for details. The code of PearMODB is available at https://github.com/tripal/.
